# Comprehensive analysis of the genome transcriptome and proteome landscapes of three tumor cell lines

**DOI:** 10.1186/gm387

**Published:** 2012-11-18

**Authors:** Pelin Akan, Andrey Alexeyenko, Paul Igor Costea, Lilia Hedberg, Beata Werne Solnestam, Sverker Lundin, Jimmie Hällman, Emma Lundberg, Mathias Uhlén, Joakim Lundeberg

**Affiliations:** 1 KTH - Royal Institute of Technology, Science for Life Laboratory, School of Biotechnology, SE-171 65 Solna, Sweden

## Abstract

We here present a comparative genome, transcriptome and functional network analysis of three human cancer cell lines (A431, U251MG and U2OS), and investigate their relation to protein expression. Gene copy numbers significantly influenced corresponding transcript levels; their effect on protein levels was less pronounced. We focused on genes with altered mRNA and/or protein levels to identify those active in tumor maintenance. We provide comprehensive information for the three genomes and demonstrate the advantage of integrative analysis for identifying tumor-related genes amidst numerous background mutations by relating genomic variation to expression/protein abundance data and use gene networks to reveal implicated pathways.

## Background

Human cancer cell lines have been an invaluable and practical resource for cancer research. The availability of genomic, transcriptomic and proteomic data on these lines is expected to further increase their utility. To this end, we conducted whole-genome and transcriptome sequencing on three tumor cell lines (A431, U251MG and U2OS) for which there is a large body of proteomics data [1]. The choice of these lines was also motivated by their origin from different lineages (tumor cell lines from mesenchymal, epithelial and glial tumors) and abundance of literature.

A431 is used as a model cell line for epidermoid carcinoma and there are currently 3,359 publications describing studies using this cell line. It was established from an epidermoid carcinoma in the vulva of an 85-year-old patient [2]. This cell line expresses high levels of epidermal growth factor receptor (EGFR) and is often used to investigate cell proliferation and apoptosis. U251MG is a commonly used glioblastoma cell line (over 1,200 published articles) established from a male's brain tissue [3]. U2OS is an osteosarcoma cell line derived from a 15-year-old female [4]. Osteosarcoma tumors arise from cells of mesenchymal origin that differentiate to osteoblasts. It is the most common form of bone cancer, responsible for 2.4% of all malignancies in pediatric patients, and its triggers are currently not known [5]. U2OS is a common choice for osteosarcoma research: 35% of the articles associated with the osteosarcoma Medical Subject Headings (MeSH) term in the PubMed database have used this cell line.

Using modern technologies, we subjected these three cell lines to genome and RNA sequencing in order to identify genomic alterations and expression of messenger and microRNAs. A review by Ideker and Sharan summarized studies that demonstrate how genes with a role in cancer tend to cluster together on well-connected sub-networks of protein-protein interactions [6]. We also earlier demonstrated that somatic mutations in a glioblastoma cancer genome produced a pathway-like pattern of enriched connectivity in the gene interaction network. Hence, in this work we analyzed functional relations between all detected somatic mutations, structural variations (altered copy number) and allelic imbalances of expression via network enrichment analysis (NEA) [7,8]. A biological pathway could be seen as an area of densely connected genes in a functional gene network. The idea of NEA when applied to cancer-related genes is that multiple key mutations (which are believed to be common in cancer genomes) could alter normal cellular programs for proliferation, differentiation, cell death, and so on, sometimes even producing quasi-pathways [9]. These altered pathways could then be detected as denser and more enriched areas and evaluated by comparing patterns formed by the same set of genes in biologically meaningless (random) networks. Either the whole group or members of such a pathway could have links to individual master switches of oncogenesis, which may themselves have not been altered.

In particular, Dutta and co-authors developed a valuable idea, according to which effects of driver genes might be seen as differential (mRNA or protein) expression of network neighbors [10]. In the current work we pursue a similar approach with the difference that we did not make any prior assumptions about modular properties of driver mutations and entirely summarized their relations to each other and important pathways. This method is the closest analog of gene set enrichment analysis (GSEA), with the important novel option of analyzing single genes against functional sets [11]. Apart from that, gene network information enables much higher sensitivity, which we demonstrate as well.

While different methods of network inference from single or two data sources have been published [12], only data integration networks have a broader scope and include multiple molecular mechanisms required for our analysis. For the highest completeness, we employed a network of functional coupling that was drawn up using the methodology of the data integration tool FunCoup [13], and then merged with curated pathways from Kyoto Encyclopedia of Genes and Genomes (KEGG), protein complex data from CORUM, and a special network from glioblastoma data. However, any state-of-the-art network is likely incomplete or does not account for a specific context and we thus complement the network analysis of direct links with analogous statistics that accounts for indirect links, that is, connections via third genes.

To enable a rigorous statistical evaluation, patterns of potential functional couplings are compared to observations in a series of randomized networks that preserve basic topological properties overall, but have no biological function. This results in probabilistic estimates for every tested hypothesis. As the analysis considers relative enrichment rather than absolute signal strength, functional patterns can be discerned in the presence of multiple spurious mutations, which are referred to as passengers. On the other hand, any computation-based gene network would have a high number of individual false edges. Again, looking at statistically significant enrichment patterns instead of focusing on particular links allows ignoring such false positive findings. Of note, a number of reports were dedicated to discovery of network structures (modules, clusters, hypothetical pathways, and so on) that could characterize pathologic conditions [10,14,15].

Here we describe, to our knowledge, the first study in which whole-genome and transcriptome data for three cancer genomes were analyzed in conjunction with data on global protein levels. First, we select genes with the potentially highest signal concentration (that is, filter them by expression values, correlation of those to genome alteration, sequence features, and so on), and subject them to network enrichment analysis to prove that both the selection criteria and NEA can bring us closer to the true sets of driver mutations in these genomes. Second, we re-analyze in the interaction network all detected copy number and single nucleotide alterations and present the most likely driver mutations within each genome. We show that passengers account for the overwhelming majority of all detected structural variations. We believe that the results presented herein provide a basis for understanding the functional interactions between the genome, transcriptome and proteome for both these highly influential model cell lines and cancer genomes in general.

## Materials and methods

### Sequencing and mapping

We sequenced six Illumina paired-end lanes for the osteosarcoma (U2OS) cell line, and five for each of the other two cell lines, glioblastoma (U251) and epidermoid carcinoma (A431). In total, there were 16 lanes, amounting to 1.23 billion paired-end reads. The data are publicly available [ERP001947] [16]. The lanes were then mapped to the human genome, hg19, using BWA [17]. BWA was run with default parameters except for: -l 25 and -k 2. With these settings, 90%, 92.6% and 88.3% of the reads were mapped for the U251MG, U2OS and A431 cell lines, respectively. Mapped lanes were then filtered on a mapping quality higher than 30 to retain only the best mappings. Reads that mapped in multiple locations, which are reported by BWA as having quality 0, were discarded. This conferred coverage of approximately 21 × for U2OS. For U251 and A431 the coverage was approximately 15 ×. In addition to the paired-end libraries, we also sequenced three mate-pair lanes, one for each cell line. After clipping adapter sequences and reverse complementing the reads, we mapped them using BWA with the same parameters as above.

### mRNA sequencing

Total RNA was extracted using the RNeasy Mini extraction kit from Qiagen (Hilden, Germany) and eluted in 50 μl of RNase-free water. The quality of the RNA was analyzed using the Experion Automated Electrophoresis Station from Bio-Rad and the standard sensitivity RNA chip (Hercules, California, US). The RNA quality indicator (RQI) was 10 for all samples. The RNA extracts were stored at -80°C. Each RNA sample was bar-coded and prepared according to Illumina mRNA-seq sample preparation and kit with the automated platform previously described [18]. The barcoded libraries were pooled together in pairs at equal concentrations and clustered on a cBot cluster-generation system using the Illumina HiSeq single-read cluster generation kit according to the protocol from the manufacturer. The pooled libraries were sequenced on Illumina HiSeq 2000 following instructions for multiplex single read sequencing and using 100 + 7 cycles. All lanes were spiked with a control library of phiX, yielding about 1% of the sequencing reads per lane. Reads were then mapped with TopHat with no quality trimming either with g -5 or g -20 [19]. The data are publicly available [ERP001948] [20].

### Functional analysis of the gene interaction network

#### Network construction

The existing global networks of functional coupling, such as FunCoup, PPI networks, the union of KEGG pathways, and so on, are known to be of high quality and relevance when applied to statistically evaluate functional relations between larger gene sets. As the network for the enrichment analysis, we predicted a human network of functional coupling using the FunCoup computational framework at a confidence cutoff for individual links defined as a final Bayesian score >7 [13]. This updated version used the latest protein-protein interactions from the IntAct database, protein expression atlas HPA [1] and sub-cellular localization data from Gene Ontology. In addition, analysis of glioblastoma multiforme (GBM) published by The Cancer Genome Atlas [21] provided data on the methylation status of about 2,000 genes, and the transcription of more than 17,000 genes; the GBM network was constructed by simultaneously profiling 147 individual tumors for genomic changes in 500 genes. This dataset provided an opportunity to reconstruct a cancer-specific network that considers the three molecular mechanisms. Using partial correlation analysis [22], we obtained a compact and highly specific GBM network of causative relations between somatic mutations, methylation, and transcription (22,990 links between 15,197 gene symbols; (manuscript in preparation). The FunCoup network was then merged with the GBM network and 79,539 curated links between 5,763 genes from the KEGG [23] and CORUM [24] databases. In total, the union contained 889,654 unique links between 18,904 HUPO gene symbols.

#### Functional gene groups for network analysis

To characterize altered gene sets by involvement into known biological processes, we compiled a list of gene membership in pathways and other gene groups of importance in the cancer context: 1) all 235 pathways presented in the KEGG database (as of 21 April 2010), including 9 cancer pathways; 2) 15 Gene Ontology terms that could be related to hallmarks of cancer [9]; 3) 13 cancer-related pathways from publications reporting on large-scale cancer genome projects; 4) gene sets of epithelial-mesenchymal transition (courtesy of S Souchelnytskyi) and tumor-specific pH-shift (courtesy of A de Milito). The list thus included 5,698 distinct HUPO gene symbols assigned to 260 gene groups (multiple membership allowed).

#### Network enrichment analysis

For two gene sets, one of which is a set of altered genes (the altered gene set (AGS)) *i *and the other a functional gene set (FGS) *j*, the confidence of functional connectivity, that is, enrichment in network connections *n_ij _*between *i *and *j*, was estimated with a z-score:

$$z=\frac{n_{ij}-{\hat{n}}_{ij}}{\sigma_{ij}}$$

where *n_ij _*is the total number of links between any genes of *i *and any genes of *j *found in the given network. In biological networks, the distribution of node degree (number of connections per gene node) follows the power law, that is, is very uneven: many nodes have few links, while few nodes have many links. Thus, the expected (mean) number and standard deviation *σ_ij _*estimates are strongly influenced by node degree compositions in particular gene sets. To make the analysis unbiased, we applied the network randomization procedure proposed by [25]. While systematically re-wiring network nodes, that is, randomly swapping edges between two nodes at a time, the procedure preserved node degrees and the total number of edges in the network. The expected mean ${\hat{n}}_{ij}$ (counted in the same way as the value of *n_ij_*) and standard deviation *σ_ij _*were learned after a sufficient number (50) of random network permutations. The default statistic counted the direct links. An alternative statistic counted links indirectly, via a shared network neighbor, that is, if there was a third gene linked to both genes in question. Under the true null, that is, in the absence of any functional linkages between gene groups, the z-scores must be normally distributed; hence, *Z *could be converted to *P*-values by a standard procedure. For both direct and indirect links in each analysis, we evaluated relevant false discovery rates by looking at the left tail of the z-score distribution (that is, the depletion side) where no significant findings were expected and, alternatively, by permutation tests on random gene sets of matching size and topological properties.

Each gene carrying a potentially damaging single nucleotide variant (SNV) was individually tested for functional relatedness to the rest of the genes with potentially damaging SNVs from the same somatic genome. Formally, we tested for violation of the null hypothesis that stated 'the individual gene is not enriched in connections with somatically mutated genes from the same line' using two different statistics (direct and indirect links); we performed 334 tests in total (2 × (57 + 51 + 54)).

### Gene set enrichment analysis

GSEA was performed on fixed-size AGS against the same FGS as described for NEA using the hypergeometric test, also known as odds ratio test [26]. The z-scores were converted to *P*-values and adjusted for multiple testing with an R function using the Benjamini and Hochberg method.

## Results and discussions

### Genes affected by structural variations and their functional implications

**Figure 1:**
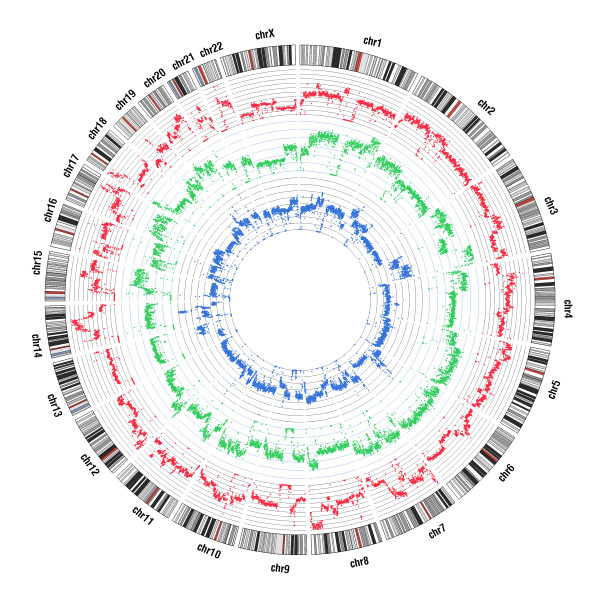
**Whole genome read coverage plots of A431 (blue), U251MG (green) and U2OS (red) cell lines in Circos format**. The coverage profile was computed for windows of 250 kb. For each cell line, the middle line corresponds to no copy number change, and data points above represent amplifications and those below represent losses. The outermost circle represents the chromosomes with cytogenetic bands.

Numerous structural variations were identified [27,28] and their breakdown is given in Table S1 in Additional file 1. In summary, we detected 1,405, 1,340 and 1,497 deletions (≥300 bases) in A431, U251MG and U2OS, respectively (Additional file 1). The depth of coverage was used to call for gained or lost regions in these genomes (Table S2 in Additional file 1) [29]. In A431, 27% of the genome was amplified but only 2% of the genome was lost (Figure 1). Similarly, the U251MG cell line gained 25% of its genome and lost around 2% (Figure 1). In contrast, an equal portion of the genome (19%) was gained and lost for the U2OS cell line (Figure 1). The U2OS cell line has lost one copy of TP53 (its expression is halved compared to other cell lines), which could influence the extent of genomic deletions [30]. U2OS also lost one copy of chromosome 13 and chromosome X, which constitute 40% of its losses (Figure S1). It also has a mis-functioning copy of ATRX due to a large deletion that removes 16 exons. Reduced levels of ATRX, which performs regulatory functions at interphase, can induce segregation defects resulting in lagging chromosomes, which could explain whole chromosome losses in U2OS [31].

**Figure 2:**
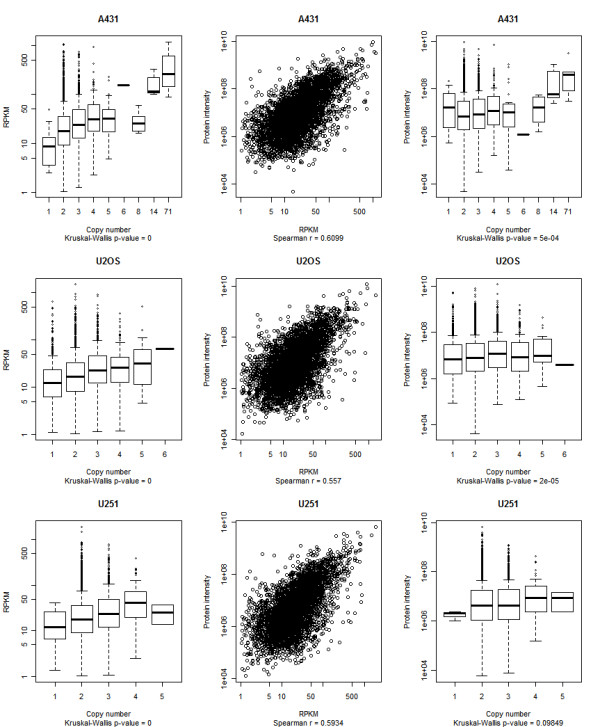
**Correlations between gene copy number, transcription, and protein abundance**. Within each cell line, correlations between the three values were estimated for the 4,554 genes that had protein intensity data. Each row shows data for one of the three cell lines. Left panels: non-parametric ANOVA with gene copy number as the factor and the RPKM values as the response (the box shows the 25th, 50th, and 75th percentiles. The length of the error bars is equal to 1.5 times the interquartile range and the quoted *P*-values refer to the Kruskal-Wallis ANOVA test). Middle panels: relationship between RPKM values and protein intensity. Right panels: same as left panels but with protein intensity as the response.

We also profiled mRNA expression in each cell line using sequencing. To investigate the extent to which changes in genomic copy number of a gene affect its level of transcription, we classified all genes according to their copy numbers. Genomic copy number changes showed pronounced effects on transcript levels: genes with high copy numbers were expressed at significantly higher levels than those with lower copy numbers (Figure 2a; *P*-value = 1e-06). The relationship between genomic copy number and protein expression was also investigated by considering protein abundance data obtained by SILAC-based mass spectrometry analysis [32] for the proteins encoded by the 4,554 most strongly expressed genes for each cell line. In keeping with previous findings [33], we observed a modest correlation between gene expression and protein abundance (Spearman's r = 0.55-0.61; Figure 2b). We then looked at the direct relationship between copy number and protein abundance. There was a positive relationship between copy number of genes and their protein abundance. The impact of gene copy number on protein levels was lower than that of mRNA expression. This is expected since pre-translational steps also modulate available transcript amounts for translation (Figure 2c; *P*-value = 5e-04).

**Figure 3:**
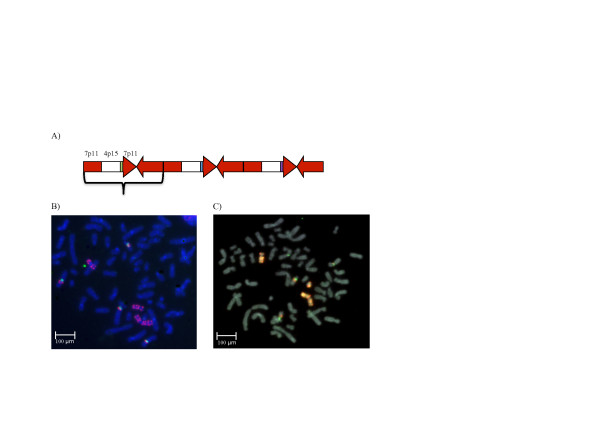
**Complex amplification of *EGFR *and *PPARGC1A *loci**. **(a) **The region within the curly bracket is the tandemly duplicated unit in reverse orientation. It contains a 639 kb region (chr7: 54,973,500-55,632,000, red arrow, carrying the *EGFR *gene) and its inverted partial duplicate that contains the 1.3 Mb region on chr4 (chr4: 22,864,000-24-249,500, white box, carrying the *PPARGC1A *gene) and shorter regions from chromosomes 1, 21 and 3 (green, blue and purple boxes). **(b) **A431 cells in the metaphase, pink probes target amplified EGFR (340 kb), green probes target the centromere of chromosome 7. EGFR is located in chromosome 7 as well as in two minute chromosomes. **(c) **The probes targeting the *PPARGC1A *locus (chr4p15) and *EGFR *are visualized together, confirming the co-localization of these two heavily amplified loci in the minute chromosomes.

A431 overexpresses EGFR and is often used as a positive control for EGFR expression. We found a complex pattern of EGFR amplification in the A431 cells using long-insert libraries (Additional file 1): a 247 kb region carrying most of the 5' end of EGFR was amplified by a factor of 154 and an adjacent 392 kb region carrying the 3' end of EGFR and two other genes was amplified by a factor of approximately 77. The chromosome section encompassing both of these regions was tandemly duplicated with its orientation reversed several times. However, the 392 kb region had been deleted in approximately half of the copies, which is why it was only amplified half as much as the 247 kb region. In cases where the 392 kb region had been deleted, it was replaced with a 1.3 Mb region from chromosome 4, which was also amplified by a factor of 77 as a result. In addition, several regions from chromosomes 1, 21 and 3 were inserted and amplified together (Figure 3a). We performed fluorescence in situ hybridization (FISH) experiments using probes against EGFR and PPARGC1A loci to locate their excess copies (Figure 3b,c; Additional file 1). In addition to its native position, numerous copies of EGFR were found in two artificial chromosomes that appear to only carry the rearranged copies of EGFR and PPARGC1A (Figure 3c). The region on chromosome 4 contains one gene, *PPARGC1A*, which is a transcriptional coactivator involved in relaying environmental signals to control the metabolic activity of cells [34]. Its normalized expression levels (reads per kilobase per million mapped reads (RPKM)/gene copy number) are similar in all cell lines (approximately 0.8). In A431, however, its amplification appears to have increased its RPKM to 56.8.

### Analysis of potential downstream effects of point mutations in all cell lines

SNVs were detected within coding genes [27] (Additional file 1). We first investigated effects of splice site SNVs on transcriptomes of the three cell lines. An in-house software package was used to evaluate the effects of splicing site SNVs on transcript structures (Additional file 1). Approximately 2,500 SNVs were found that may potentially affect splicing in each cell line; after applying several filters, around a dozen were identified as being potentially damaging and only two of these were validated by reference to mRNA data (Table S3 in Additional file 1). *APIP *was found to undergo alternative splicing in U251, probably due to a homozygous splice site SNV (chr11: 34905054_G/C) at the upstream splice site of exon 6 (Figure S2a in Additional file 1). This mutation causes the sixth exon to be skipped without shifting the reading frame. An aberrant transcription of the proto-oncogene *FES *was detected (Figure S2b in Additional file 1) in U2OS cells, which is missing the first 15 exons (which contain the regulatory region of its protein activity), leaving only 4 expressed exons. FES without its regulatory part has also been observed in lymphoma and lymphoid leukemia cell lines [35], and appears to be produced from the same transcript as we found in the U2OS osteosarcoma line in this work. FES expression has been found to correlate with tumor growth and metastasis [36] and it is likely that the short transcript variant observed in U2OS was involved in carcinogenesis.

We also assessed allelic imbalances in the expressed genes by comparing individual SNV frequencies at the DNA and RNA levels (Additional file 1). Genes carrying SNVs that were heterozygous at the DNA level but homozygous in RNA transcripts were considered allelically imbalanced. We detected 17, 6 and 10 such genes in A431, U251MG and U2OS, respectively (Table S4 in Additional file 1), and only one of them (*NDN*) is imprinted [37]. In A431, several transcription factor genes as well as *HDAC8, SMARCA1 *and *BCLAF1 *were expressed from only one allele. *MAP2K3 *was allelically imbalanced in both the U2OS and U251MG cell lines.

We then looked at the non-synonymous SNVs in these genomes. In order to enrich those involved in tumor maintenance, we applied filters based on their heterogeneity and common polymorphisms (Additional file 1). We then predicted their protein-level effects using PolyPhen to filter out those with no obvious potential to cause a functional change on the protein [38]. This left us with 57, 54 and 51 genes carrying SNVs that were likely to be damaging to protein function in A431, U251MG and U2OS, respectively (Table S5 in Additional file 1).

Cancer state is likely to be the result of a set of functional mutations in key genes that perturb relevant gene networks at multiple points [9,39]. To identify such cooperative actions of mutations, we used NEA aiming to find the most likely key genes for each cell line, that is, the impaired genes that contributed to the onset and/or maintenance of the rapid proliferation state. To this end, we evaluated network connections between the genes impaired via SNVs within each cell line. In the A431 cell line, 8 of 57 potentially impaired genes were strongly connected to other genes within the same set; the corresponding numbers for the U251MG and U2OS lines were 12 and 7, respectively (false discovery rate (FDR) <0.10; Table S6 in Additional file 1). One example is *PKMYT1*, a gene that carries a heterozygous SNV that is predicted to be damaging (NP_004194_E179G, PolyPhen FDR = 0) in U2OS cells. This mutation is at a conserved residue within the catalytic domain of the protein [40]. NEA indicated that this mutation was only directly linked to one other damaging somatic mutation in U2OS - a mutation in carbamoyl phosphate synthetase II (CAD). However, analysis of indirect links (that is, those via shared neighbors) revealed significant relationships between *PKMYT1 *and the rest of the U2OS somatic mutation set (790 links compared to 406.4 expected by chance, NEA z-score = 19.21). Again, the majority of such links (Figure S3 in Additional file 1) led to CAD through BMP2K and CDK2 (502 links), nuclear protein NUP93 (72 links), the WD repeat and HMG-box DNA binding protein WDHD1 (54 links), and the DNA primase PRIM2 (53 links). Collective actions of these heavily connected impaired genes could produce alterations in associated pathways such as cell cycle regulation [41,42].

### Context-dependent meta-analysis of impaired genes in the three cell lines

**Figure 4:**
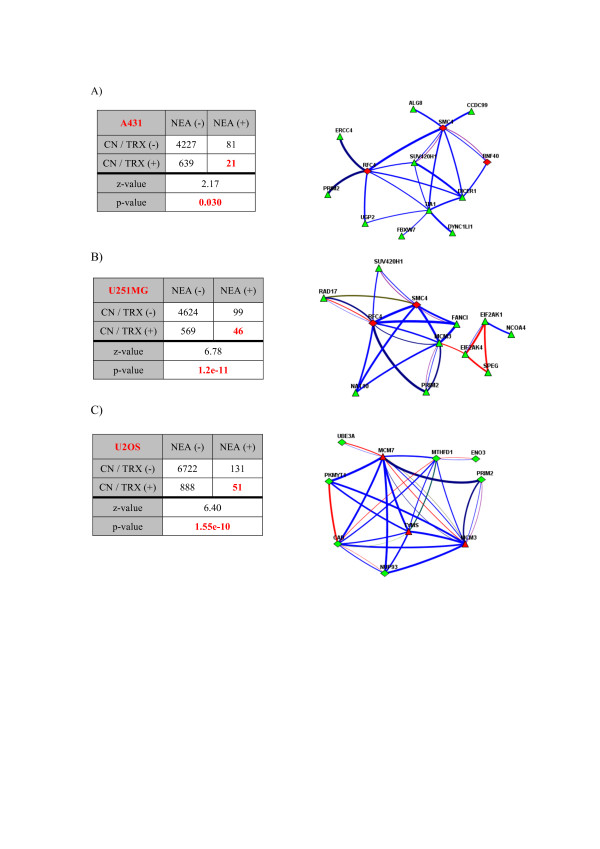
**Contingency tables for genes identified using two different filtering schemes**. **(a) **A431, **(b) **U251MG and **(c) **U2OS cell lines. CN/TRX (+) denotes genes for which the average Spearman coefficient over all three cell lines for the relationship between copy number and transcription is >0.8. NEA (+) denotes genes exhibiting enriched connectivity to genes carrying damaging mutations in the same cell line (NEA z-score >1.96). These criteria were selected to optimize the 'sensitivity/specificity' trade-off after having considered several alternatives (Figure S4 in Additional file 1). In the network diagrams in the same cell line order, red triangles denote CNA genes coupled with more than five links to genes carrying damaging SNVs in the same line, denoted as green diamonds. The color scheme for the connections is: red lines for physical protein interaction, blue lines for mRNA co-expression, green lines for protein co-expression, purple lines for sub-cellular co-localization, khaki lines for coherence of Gene Ontology annotation, deep bluish green lines for links in a KEGG pathway, and deep blue lines for known members of the same complex.

Somatic mutations in key genes are central to the initiation of cancer state and concurrent copy number alterations can contribute to further progression and maintenance of the rapid proliferation state. Specifically, the affected genes can facilitate subclonal expansion - for instance, by conferring a growth advantage or enabling cell death evasion [43]. The resulting cancer circuitry thus involves the concerted action of multiple genes that have undergone copy number or point mutations; that is, the formation of the circuitry is independent of the mechanism by which the damage to each gene was sustained. Importantly, whether a novel mutation/structural variation will be advantageous for the rapid proliferation is defined by its interactions with the rest of the (pre-existing) mutations and the transcriptional landscape. To this end, we investigated functional relations between genes affected by SNVs, allelic imbalance or copy number alterations. There were more than 3,000 copy number-altered (CNA) genes per cell line. Obviously, most of these did not contribute to the rapid proliferation state. To identify genes with a significant impact, we assumed that the transcript levels of such genes would mirror the changes in their copy number, as would the levels of the corresponding proteins. We therefore looked at the correlations between expression/protein abundance and the copy number of every gene across the three cell lines to filter out CNA genes that do not affect transcript or protein levels and are thus less likely to be involved in achieving rapid proliferation. To control for the potentially high FDR in this correlation analysis (due to the small number of cell lines considered), we performed permutation tests on the full CNA gene lists and recorded the log of the ratio of the observed correlations to those obtained from the permuted list (Figure S4 in Additional file 1). This highlighted genes with true correlations between their copy number, mRNA expression, and protein abundance values (the latter set of correlations was weaker than the former, as expected). Genes with structural variation in more than one cell line and with higher RPKM values yielded lower FDRs (Figure S5 in Additional file 1). However, even after the application of this filter, the FDR is likely to be high, leaving hundreds of false positives in the pool for consideration. Network analysis was therefore performed to exclude genes that had undergone copy number changes but are irrelevant within the context. We considered the functional interactions between a single gene from the CNA gene set and the much more strongly delineated (around 50 genes per line, as described above) set of impaired genes due to SNVs, so that the latter could serve as a reference set. NEA z-score thresholds of increasing stringency (the z-values ranged from 1.64 to 6.00, corresponding to *P*-values of 0.1 to 0.000001 and FDR values of 0.2 to 0.01 in the network analysis) were applied in conjunction with the expression and correlation criteria described above. The fractions of CNA genes affecting expression levels with and without functional couplings (that is, with low or high NEA z-scores) were compared to those for CNA genes that did not affect expression, or not significantly expressed at all (low RPKM). Remarkably, the latter group manifested much lower fraction of NEA-positive genes at any significance threshold (two- to four-fold; Figure S6 in Additional file 1). Although neither method and criteria set had perfect sensitivity, the final analysis was performed using CNA gene lists for which the mean correlation coefficient between copy number and expression/protein abundance was above 0.8 and which yielded NEA z-scores above 1.96, which corresponded to a FDR of less than 0.1 (Figure S6 in Additional file 1). Using these criteria, we identified 21 CNA genes from A431 that are likely to be functionally related to damaging SNVs; the corresponding numbers for U251MG and U2OS were 46 and 51, respectively (Figure 4; Table S7 in Additional file 1). Figure 4 displays network relations between two or three most connected CNA genes and respective SNVs of the same cell line. Remarkably, the network links connecting CNA genes and their interactors were mostly based on mRNA expression analysis (blue lines in Figure 4). In theory, copy number alterations should act through transcription, and respective genes should produce functional relations via transcription, which can then be seen in the general context network we employed. Hence, in this case we likely observed a true case of copy number alterations interacting with SNV-impaired genes. Moreover, we detected a common subnetwork (Figure S7 in Additional file 1) when we combined impaired genes from the three cell lines, although only parts of it were active in each cell line.

We then looked at the overlap with and interactions between our affected gene sets and a comprehensive list of cancer-related genes generated by Ding *et al. *(referred to as the Ding-set) [44]. SNV-impaired genes in U2OS and U251MG were significantly enriched in terms of NEA with the Ding-set but those from A431 were not. All lists manifested some enrichment against KEGG cancer pathways, but only the U251 cell line was strongly associated with these pathways. The other two only had significant z-scores against small and non-small cell lung cancers as well as prostate and bladder cancer, whereas U251 was enriched with respect to all of these and ten other cancer pathways. However, as a final test of CNA being a driver mutation, we present a context-specific analysis: a NEA of individual CNAs versus the filtered SNV gene sets of the same cell line (Table S7 in Additional file 1). This analysis is analogous to the 'SNV gene versus SNV gene set' analysis described above (Table S6 in Additional file 1). Figure S8 in Additional file 1 shows the case for a specific SNV-impaired gene, *MCM3*, in U251 and interacts with several genes in cancer pathways as well as with other SNV-impaired genes in the same cell line.

**Figure 5:**
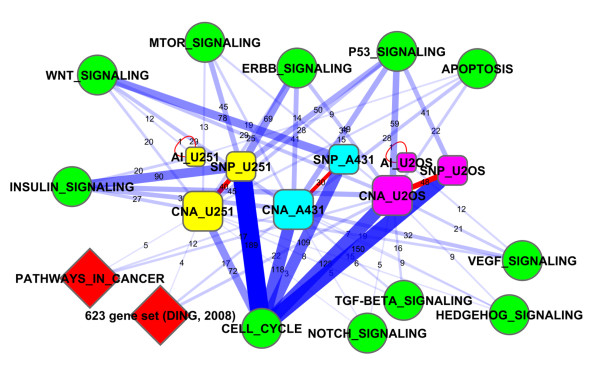
**Mapping of genomic alterations in three cell lines to most basic signaling pathways from the KEGG database, KEGG cancer super-pathway 05200, and the group of 623 genes associated with cancer by Ding *et al. ***[42]. Yellow boxes, U251MG; blue boxes, A431; purple boxes, U2OS. A single line summarizes the network connections between each pair of gene sets, with the line's width reflecting the number of links in the global network connecting individual genes from the two functional gene sets (3...189). Only relations significant by NEA are shown (*P*-value <0.05, FDR <0.10). Edge opacity and edge width reflect the number of individual gene-gene links behind the relation (also printed in brown at each edge). Mapping between experimentally determined gene sets of cell lines is highlighted in red. AI, allelic imbalance; MTOR, mammalian target of rapamycin; TGF, transforming growth factor; VEGF, vascular endothelial growth factor.

We also investigated the connectivity of each individual CNA gene to cancer-related pathways, including apoptosis, the cell cycle, and the p53 pathway. Thirty-six CNA genes displayed enriched connectivity to these pathways (at least 5 links, z-value >2) in the A431 line; the corresponding numbers of genes in the U251MG and U2OS lines were 9 and 47, respectively. Twenty-seven CNA genes were affected in more than one cell line and this overlap was stronger than that between the unfiltered CNA gene sets (*P*-value = 0.031). Fifteen cancer pathways defined in the KEGG database were significantly enriched in terms of connections to individual CNA genes from each cell line (ranging from 3 to 40 genes per pathway and cell line). Finally, we merged the three major classes of genomic alterations (copy-number changes, SNVs, and allelic imbalances) from each cell line and used network analysis to demonstrate that all of these gene classes cooperate in cancer-related activities (Figure 5), that is, there was significant network enrichment with regard to cancer-specific gene sets, apoptosis, TP53, major signaling cascades, cell-cycle and DNA-repair pathways and interactions with one another. Importantly, alterations of the three variation classes also had functional relations to each other within the cell lines (indicated by red lines between SNV and CNA and self-loops of allelic imbalance (AI) in Figure 5).

## Conclusions

In this study, we performed whole-genome, mRNA sequencing and analyses for three tumor cell lines. The expression and proteome profiles of these cell lines have already been investigated and fair correlations were shown between RNA expression and protein levels [32]. We here incorporated whole genome data such as gene copy number and DNA variation profiles of these cell lines to perform an integrative analysis and discover impaired genes and pathways. Genes with elevated copy numbers were identified in all three of the cell lines considered, giving more than 3,000 genes with copy number changes. The expression levels of each such gene and the abundance of their corresponding proteins were then used to identify genes that were likely to contribute to the maintenance of the cancer state. This analysis narrowed the list of affected genes from thousands to a few hundred per cell line, demonstrating the utility of using DNA variation together with expression data. The cell lines used in this work have different origins so our cross-correlation analysis based on the assessment of copy number-dependent expression could potentially generate false negatives or positives due to some genes being differently regulated in the different cell lines. However, we assume that while these cell lines may retain some aspects of their original identities, the extent of cell-specific changes in the expression of genes in common pathways such as cell cycle regulation, DNA replication or apoptosis have much less impact than those induced by copy number changes.

While the reduction in the number of candidate genes achieved by applying the first filter was substantial, it was not sufficient by itself because the list still contained many passengers. To address this issue, we assumed that 1) cancer is more likely to be maintained by a set of interrelated mutations that alter cellular processes at multiple points than by the effects of a single mutation, and 2) proliferative benefit conferred from an alteration can depend on already existing mutations or structural variations. We therefore focused on CNA genes that exhibited functional links to genes impaired by SNVs in the same cell line. In conjunction with the first filtering step based on the expression correlations with copy number changes, this second filter afforded significant improvements, reducing the number of putative genes contributing to rapid proliferative state to around a few dozen genes per cell line, all of which exhibited enriched connectivity to major signaling, cell division and cancer-specific gene sets. Despite the low overlap between the altered gene sets for each cell line, the network analysis demonstrated that their cancer-related functionality was cooperative, which we detected at both the pathway and global-network level.

Traditionally, novel experimentally determined AGSs are characterized by significance of overlap (amount of shared genes) with known functional gene sets. This method is generally called gene set enrichment analysis. To illustrate superiority of our NEA, we directly compare analyses from GSEA and NEA in Figure S9 in Additional file 1. Only four of all 420 analyzed AGS-FGS pairs showed a significant GSEA overlap (each case was based on two shared genes) when NEA did not detect enrichment. The number for the opposite case (NEA+, GSEA-) was 75, and 18 pairs were detected by both methods. In addition, grounding a GSEA result on two or three genes would not be robust, whereas NEA results are usually based on tens or hundreds of network links. Of note, these comparisons were only possible on AGS as sets of multiple genes, while single gene analysis against FGS is a unique feature of NEA.

Cancer cells modulate their metabolism to switch from mitochondrial to glycolic metabolism despite the presence of sufficient oxygen levels to support the former; this is known as the Warburg effect [45]. In A431 cells, lactase dehydrogenase (LDHA) levels are elevated (RPKM of 751, no gain or loss) which suggests heavy use of glycolic metabolism. The gene *PPARGC1A*, expressed strongly in normal tissues with high-energy demands, including cardiac tissue, brown fat, and the central nervous system [46-48], is heavily amplified in these cells. It is a master co-activator for mitochondrial biogenesis, which might suggest utilization of oxidative phosphorylation by A431 cells. The functional implications of this amplification are currently being assessed.

We also detected several allelically imbalanced genes and most of these genes did not have any copy number changes and/or damaging SNVs. One special case was necdin (*NDN*), a gene that is typically maternally imprinted and is only expressed in the brain and placenta [49]. *NDN *is highly expressed in the U2OS cell line but not in U251 or A431. Previous comparisons of H3K36me3 gene expression patterns between osteoblasts and U2OS suggested that it is not expressed in osteoblasts [50]. Maheswaran *et al. *[51] showed that overexpression of TP53 causes rapid apoptotic cell death in U2OS cells. However, transfection of U2OS cells with necdin together with TP53 inhibited TP53-induced apoptosis [52]. A single functional copy of *TP53 *is present in U2OS cells. This suggests that U2OS cells may evade apoptosis *in vivo *due to their constitutive expression of NDN together with reduced expression of TP53.

We also looked at splice-site SNVs and detected numerous splice-site SNVs that could cause improper splicing. Only a few were supported by RNA sequencing data, which suggests that the splicing mechanism is fairly robust, in keeping with previous findings [53].

This study demonstrates that the combined analysis of genomic and transcriptomic data can provide a better functional understanding of the mutational landscape of cancer genomes than can be obtained by considering either one of these sources in isolation. The combined analysis of genomic variation and expression datasets enabled us to distinguish between variants contributing to rapid proliferation and those that are passengers. The mutational landscapes of cancers are highly variable; few shared mutations but numerous private mutations even among similar ones [54,55]. Our method could be particularly beneficial in these scenarios since it evaluates each mutated gene within its biological context to reveal impaired functional couplings to cancer-related genes that have themselves not been altered. Moreover, the analyses over global gene and protein networks enabled us to uncover relations between alterations that drive/are driven by expression and those constitutively present in the cell but mis-paired via damaging mutations. As an example, a very recent study profiled 947 independent cancer cell lines and provided information on the copy numbers and RNA expression profiles of their genes [56]. Applying the combined analysis reported herein to these cell lines could provide valuable insights into their impaired pathways and related anticancer drug sensitivity.

## Abbreviations

AGS: altered gene set; CNA: copy number-altered; EGFR: epidermal growth factor receptor; FDR: false discovery rate; FGS: functional gene set; FISH: fluorescence *in situ *hybridization; GSEA: gene set enrichment analysis; KEGG: Kyoto Encyclopedia of Genes and Genomes; NEA: network enrichment analysis; RPKM: reads per kilobase per million mapped reads; SNV: single nucleotide variant.

## Competing interests

The authors declare that they have no competing interests.

## Authors' contributions

JL and MU initiated the study. PA led the project, data processing and analysis and wrote the manuscript. AA performed network biology and contributed to writing the manuscript. PIC performed mapping and data processing. LH performed RNA-SEQ data analysis. SL prepared the mate-pair sequencing libraries. BWS and JH prepared the total RNA sequencing libraries. EL provided protein abundance data. All authors read and approved the final manuscript.

## Supplementary Material

Additional file 1 **Supplementary methods, Supplementary Figures S1 to S9, and Supplementary Tables S1 to S7**. Click here for file
