# Supplementary material for: Comprehensive analysis of the genome transcriptome and proteome landscapes of three tumor cell lines
Source: Genome Med. 2012 Nov 18;4(11):86. doi: 10.1186/gm387 (PMC3580420; doi:10.1186/gm387)
Supplement: Additional file 1 — Supplementary methods, Supplementary Figures S1 to S9, and Supplementary Tables S1 to S7. [file gm387-S1.PDF]

SUPPLEMENTARY MATERIALS

SUPPLEMENTARY METHODS

Cell Culture

Three human cell lines; the osteosarcoma cell line U-2 OS (ATCC-LGC, Middlesex, United Kingdom), the glioblastoma cell line U-251MG (Prof. Bengt Westermark, Uppsala University) and the epidermoid carcinoma cell line A-431 (DSMZ, Braunschweig, Germany) were cultivated at 37°C in a 5% CO2 environment in media suggested by provider. For RNA extraction, each cell line was cultivated in four replicates and the cells were harvested during log-phase growth (60–70% confluency).

Long-Insert Mate Pair Libraries

The A431 long-insert library was prepared following to the Illumina provided protocol for preparing mate pair libraries v2 with minor deviations, a Covaris S2 instrument with blue miniTubes was used to shear the DNA to a size of approximately 3000 bp, NEBnext End Repair Module (New England Biolabs) to repair the fragments, GelRed (Biotium) to stain the agarose gel during size selection, Freeze N' Squeeze spin columns (BioRad) were used for agarose gel extraction, and repair and biotinylation was performed after size selection.
In brief, 8 µg of genomic was sheared, end-repaired (NEB), size selected by agarose gel excision, biotinylated, circularized by blunt ligation, exonuclease treated to remove non-circularized fragments, fragmented using Covaris S2, purified using streptavidin coated magnetic beads, end-repaired, A-tailed, sequence adapter ligated and PCR amplified. All steps were performed using the reagents supplied or referenced by Illumina, if not stated otherwise.
The U2OS and U251 mate pair libraries were prepared using the "paired end library preparation method" for 3 kb libraries from Roche/454 until circularized, after that they were prepared as the A431library. In brief, 5 µg of genomic DNA was sheared to approximately 3000 bp and end polished, ligated to the Roche/454 mate pair adapters (supplied by Roche), size selected using AMPure beads (Beckman Coulter), the adapters were filled-in, and the ligated DNA was circularized using Cre-recombinase and exonuclease treated. All steps were preformed using enzymes from New England Biolabs following the protocol from Roche/454.

Detection of Allelically Imbalanced Genes

Only SNVs supported by DNA were included in the analysis. SNVs were then filtered on having a frequency between 0.4 and 0.6 in DNA, a frequency lower than 0.01 or higher than 0.99 in RNA, a coverage of at least 10 in both DNA and RNA and that the corresponding gene only has homozygous SNVs in RNA. In order to call a gene allelically imbalanced, at least two supporting SNVs or one SNV for which >99% or RNA reads carried the alternative allele (since mapping bias is more likely to be acting in favor of the reference allele) were required (*48*).

Splice-site SNVs

Splice-site SNVs that detected within expressed genes (top 25th percentile) with frequency 0.45 were analyzed. Common SNVs were excluded from the analysis (HapMap CEU frequency ≤ 0.05). Putative effected transcripts were manually inspected using Avadis NGS genome browser. Two separate TopHat mappings (option –g5 or –g20) were used since we found that for some genes using either setting resulted in more accurate alignments.

Copy Number Detection

Copy number alterations and translocation events were called within AVADIS, which uses an adapted version of the PEMer software package. Minimum five pairs were required to support a

variant, minimum heterozygosity is set to 0.2. To call for copy number changes based on read coverage, FREEC was used with default settings, with ploidy setting to 2.
For finding gene fusions, we analyze the RNA-SEQ single-end data. We perform BWA style quality trimming with a quality value of 20 and a minimum length requirement of the resulting reads of 60 bp. Subsequently we split each read to create a paired end library. We do this by keeping 25 bp from each side. Furthermore we reverse complement the 25 bp of the left side, to facilitate pairing by the aligner. After mapping to the genome with BWA, pairs with insert sizes higher than 100 kb and mapping quality greater than 20 are extracted and reported to a separate file. This file is then sorted and collapsed to contain only on occurrence of a fusing event with a integer value indicating the number of supporting pairs.

SNV Detection

SNV were called using AVADIS NGS with the following settings: minimum decibel score 50; heterozygosity 0.0010; ignore reference locations with variants below 2; minimum coverage 10; do not use mapping quality if less than base quality. The Avadis NGS SNV caller is an adaptation of the MAQ SNV calling algorithm (*49*). SNVs were annotated within AVADIS NGS against RefSeq database (release 48). The SNV list was exported and filtered on SNV type (e.g. nonsynonymous coding, intronic, intergenic etc.). In AVADIS, unlike MAQ, MNPs are called by grouping adjacent SNVs together. AVADIS also extends MAQ to calling deletions and implements another algorithm for calling insertions. In total, we detected 3,250,932, 3,153,394 and 3,194,995 SNVs in A431, U2OS and U251, respectively (Table S1).
Non-synonymous coding SNVs were filtered on minimum fraction of variant reads 0.45 and HapMap CEU frequency ≤ 0.05. PolyPhen-2 (*20*) was used to predict the effect of non-synonymous coding SNVs, the output was then filtered on FDR ≤ 0.05. In order to exclude SNVs in unexpressed exons, SNV calls were filtered on RNA base coverage ≥ 5 (Table S3).

Fluorescence in situ hybridizations

The FISH probe for EGFR is purchased from Kreatech (cat. No. KBI-10702) and probes for 4p15 locus (PPARGC1A) was designed and produced by Kreatech. Briefly, slides were denatured in 70% Formamide / 2x SSC , pH 7.0 at 72 C. They were dehydrated in ice cold (-20C) 70%,90%, and 100% ethanol for 2 min each and air dried. Probe mix were denatured at 90C for 10 min then to denatured slide, covered with parafilm. The slide was incubate overnight at 37C. Parafilm was removed and slides were washed in 50% Formamide at 42 C 4 times and 3 min each, then in 2xSSC 42 C 4 times 3min each, then in 4X SSC/0,05% TRITONX-100 at room temperature for 5 min and lastly in PBS for 30 seconds. The slide was dehydrated in 70% 90% or 100% ethanol 1 min each. Then vectashild with DAPI (VectorLabs, cat. no. H1200) was applied.

SUPPLEMENTARY FIGURES

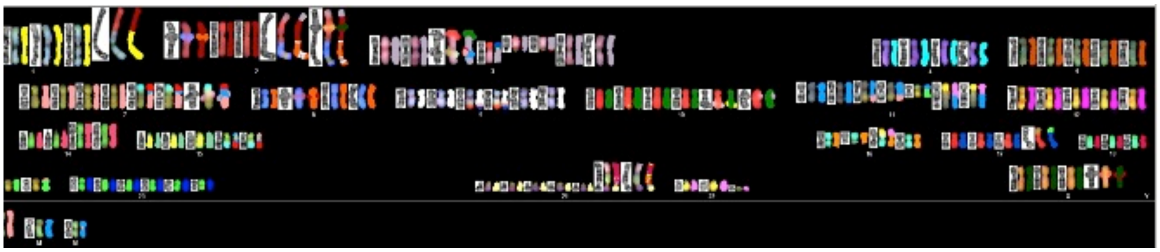

A431

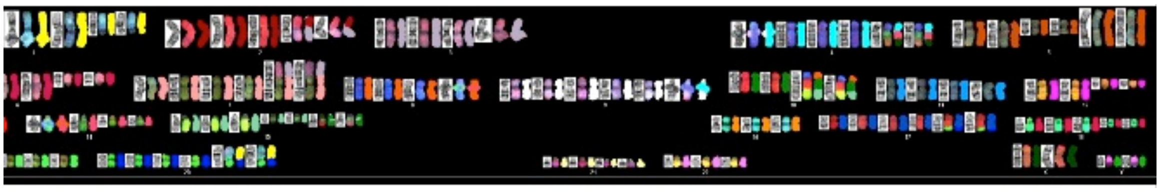

Figure S1. Sky profiles of A431, U2OS and U251MG cell lines. A431 is pentaploid

U2OS

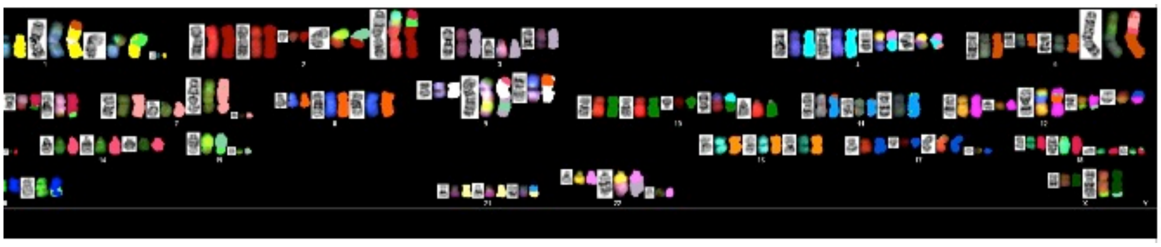

(chromosomal modal number 118). U251MG was a mix of triploid and pentaploid subclones. U2OS was mainly triploid with a small number of pentaploid subclones. U2OS displayed a mosaic karyotype with high number of non-clonal abnormalities suggesting genomic instability and ongoing evolution of this cell line.

U251MG

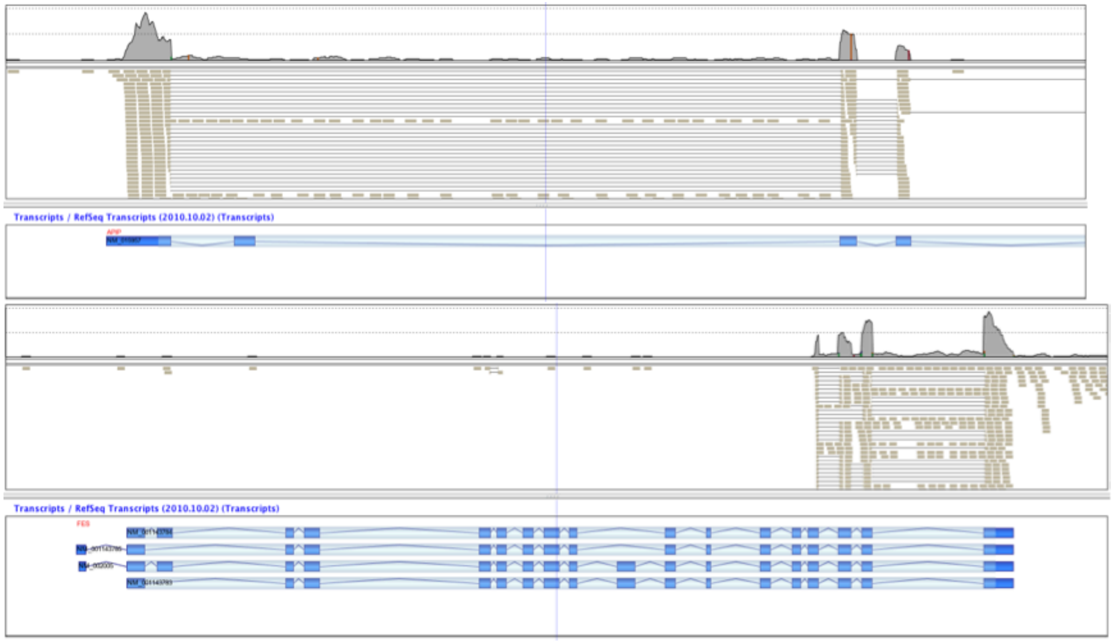

Figure S2. A) RNA read mappings towards the 3' end of APIP in U251. The second last exon exon is skipped. (chr11:34,903,014-34,911,845). B) RNA read mappings for proto-oncogene c-FES in U2OS in AVADIS. The first 15 exons are not transcribed. (chr15:91,426,800-91,440,137).

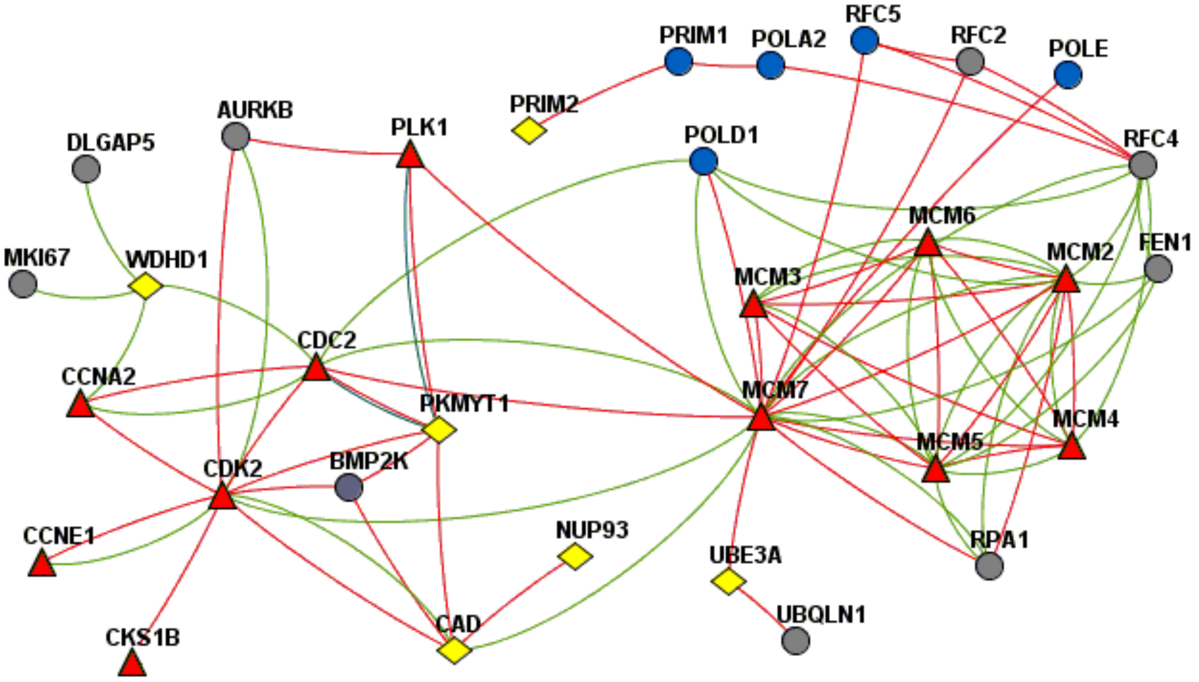

Figure S3. Common circuitry of somatic mutations in U2OS that involve known cancer-related genes and pathways. Genes labeled in yellow are mutated in U2OS, genes shown in red are involved in cell cycle regulation, and those shown in blue are RNA polymerase subunits. For the sake of simplicity, most of the links connecting U2OS somatic mutations are omitted by the FunCoup sub-network query algorithm. The only links shown are those whose existence is supported by KEGG, protein-protein interactions, and correlations of protein expression. The line types as well as line thickness (indicating confidence) are not shown for simplicity.

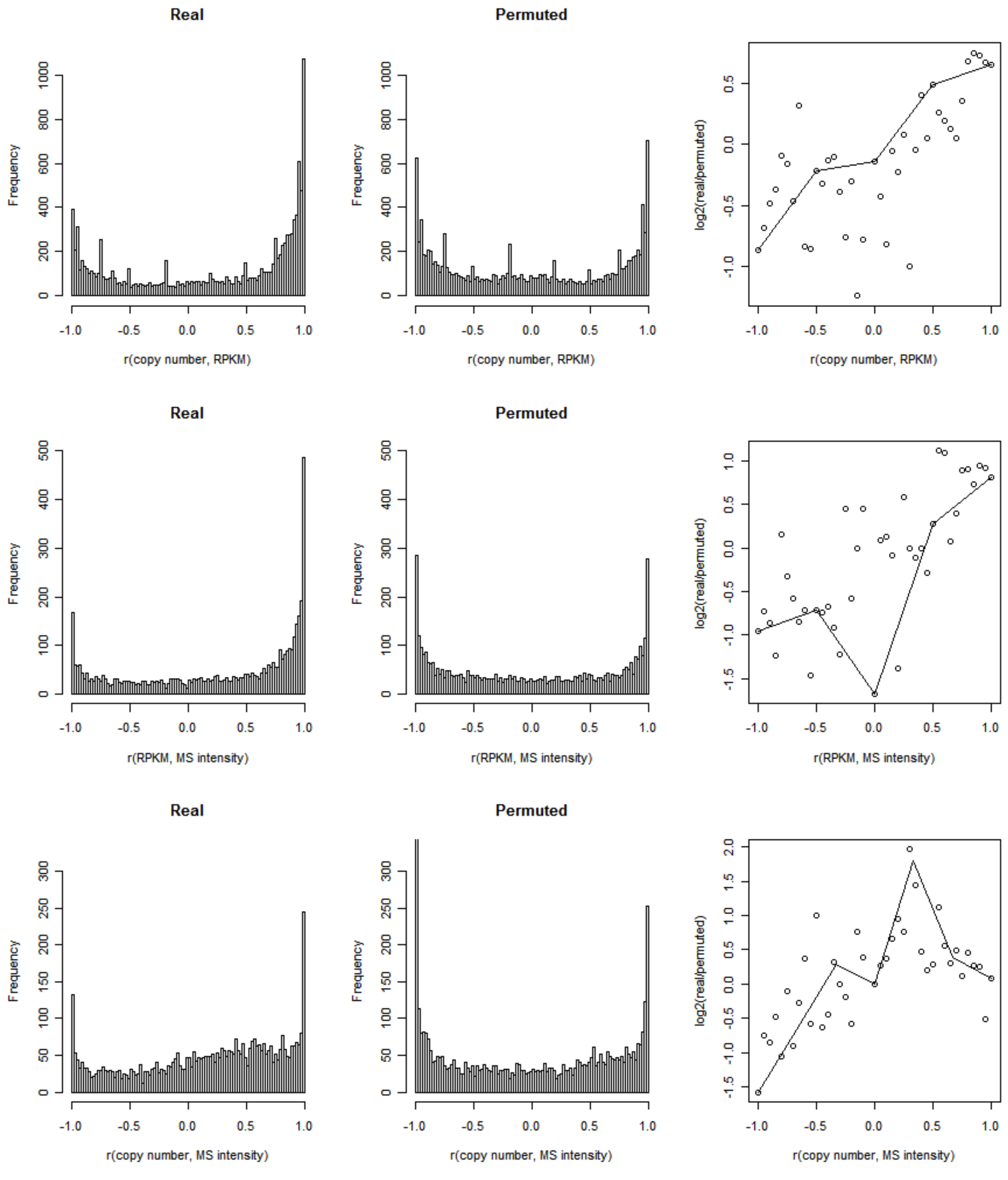

Figure S4. Distributions of Pearson linear correlation coefficients between 1) gene copy number and RPKM values, 2) gene copy number and protein product MS intensity, and 3) RPKM and MS intensity are compared between real and double-permuted profiles across the three cell lines. The last column estimates rate of true discoveries as enrichment real/permuted.

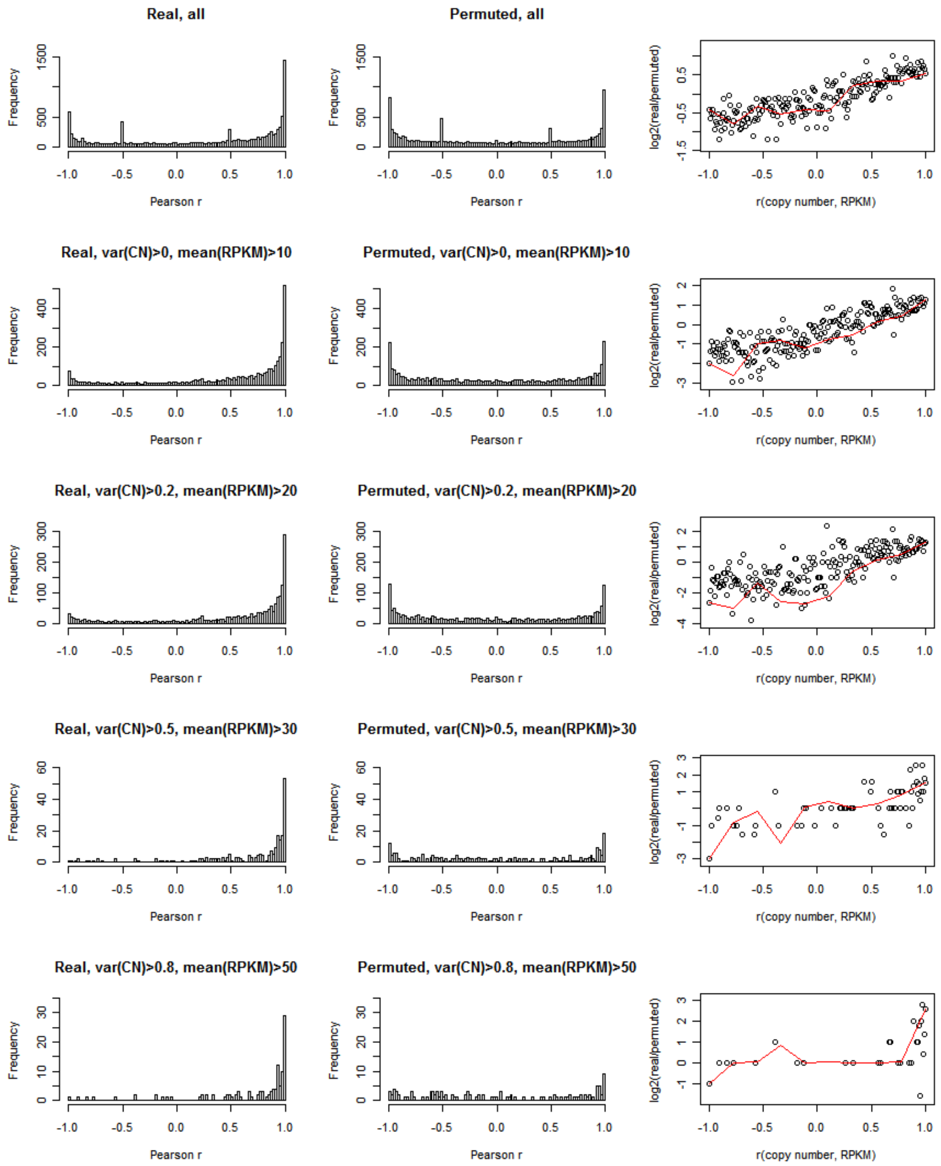

Figure S5. Distributions of Pearson linear correlation coefficients between gene copy number and RPKM values are compared between real and double-permuted profiles. The correlation analysis on profiles of just three cell lines (***n***=3) would suffer from low sensitivity and high false discoery rate (first column). To evaluate the latter, permutation tests were performed on the profiles of each genes affected by structural variation in at least one line (second column). As expected for Pearson linear correlation coefficient on small ***n*** values, fractions of genes with very high and very low correlation coefficients were large on the permuted profiles. Although, unlike of the real profiles, they were symmetrical (equal fractions close to -1 and 1) which was counter-intuitive. The fraction close to 1 on the real profiles was relatively much higher. This allowed estimating the rate of true discoveries as log ratio real/permuted (last column). First row: all SV-detected genes. Other four rows: genes that passed filtering criteria of growing stringency by transcript abundance (RPKM) and presence of copy number variation for the gene within our three cell lines (detected via variance *var(CN)*).

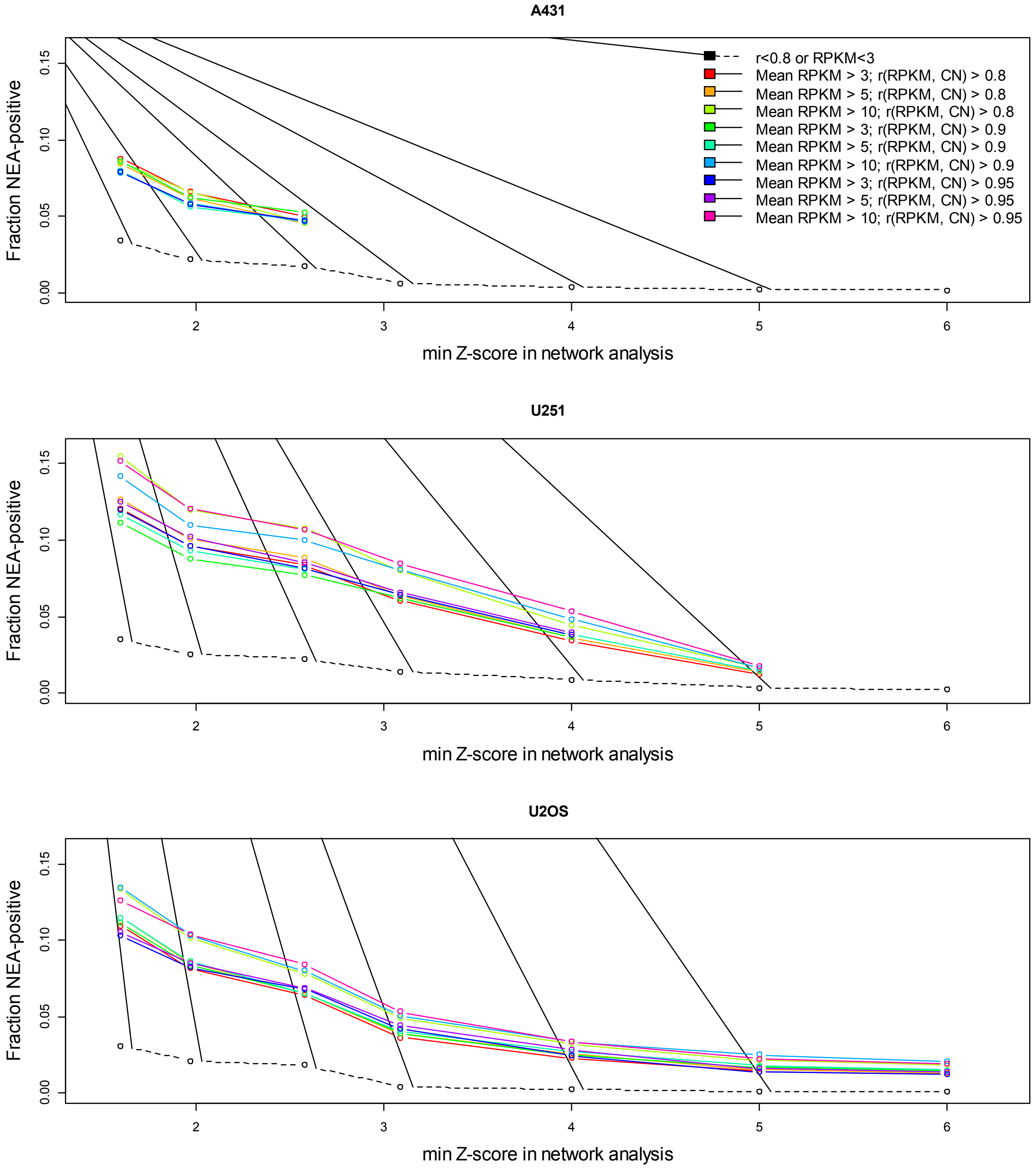

Figure S6. Fractions of copy number-altered genes that manifested network enrichment to sets of somatic mutations (SNV) from the same cell line, stratified by RPKM value and degree of correlation "CN vs. expression". Each color line continues while there is statistically significant difference (hypergeometric test, p-value<0.01) between its point and the respective point of the dotted black line.

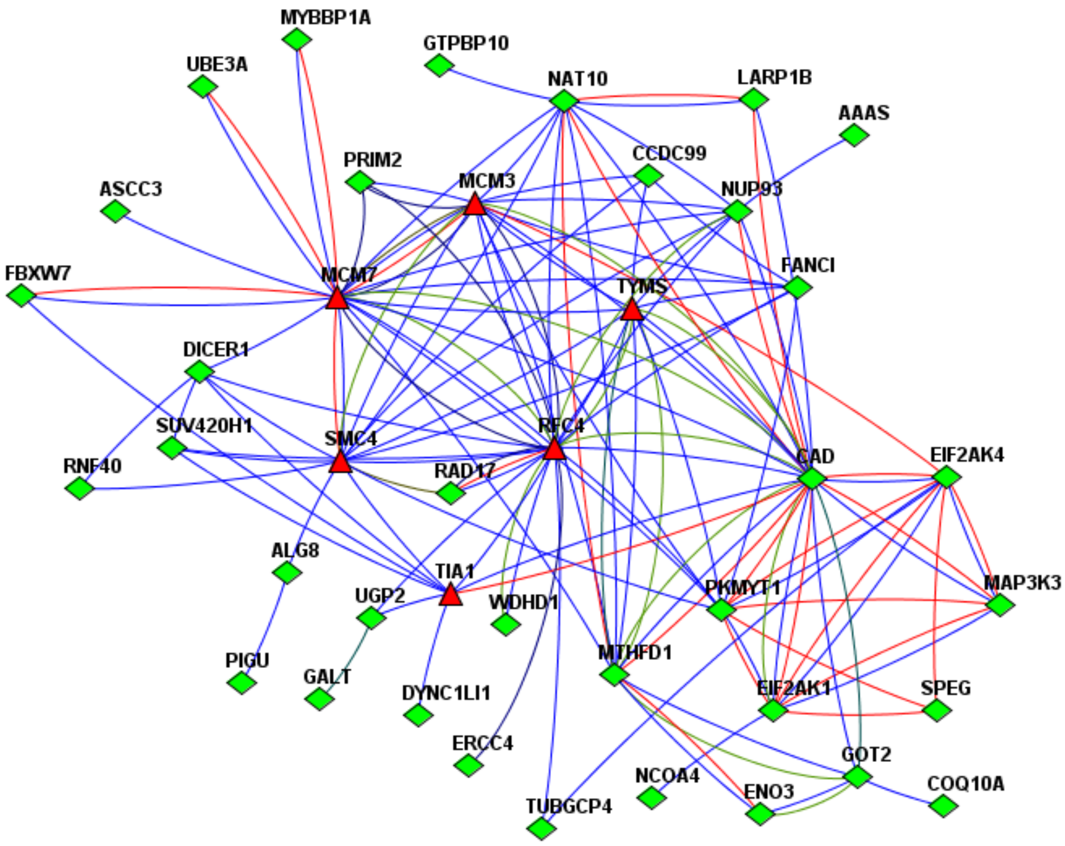

Figure S7. CNA genes and damaging SNVs most highly scoring in network analysis, combined in a single FunCoup network view. The six genes with altered copy number (same as in Fig. S8) are connected to different, cell-line specific, sets of damaging SNVs but still produce a coherent pattern in the general interactome. Green: genes with damaging mutations in three cell lines; Red: copy number-altered genes of the same lines (SMC4 and RFC4 are CNA in both A431 and U251). Coloring scheming for the connections are: red lines for physical protein interaction, blue lines for mRNA co-expression, green lines for protein co-expression, HPA). Lines denoting other available evidence and line thickness (indicating confidence) are not shown for simplicity.

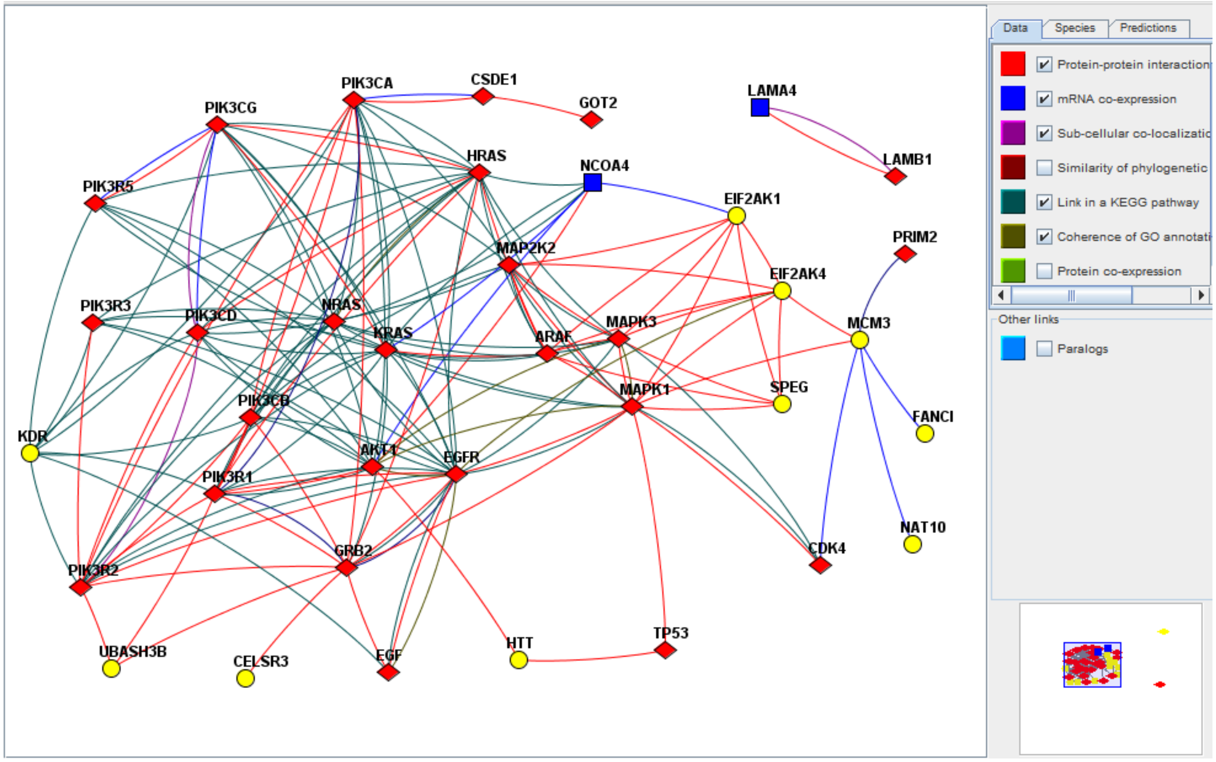

Figure S8. Interaction network of MCM3, which carries a damaging SNVs: genes with damaging mutations were linked to each other as well as to genes in cancer pathways. Yellow: genes with damaging mutations in U251; Red: members of KEGG cancer pathways; Blue: genes shared by the above groups. Line thickness (indicating confidence) not shown for simplicity.

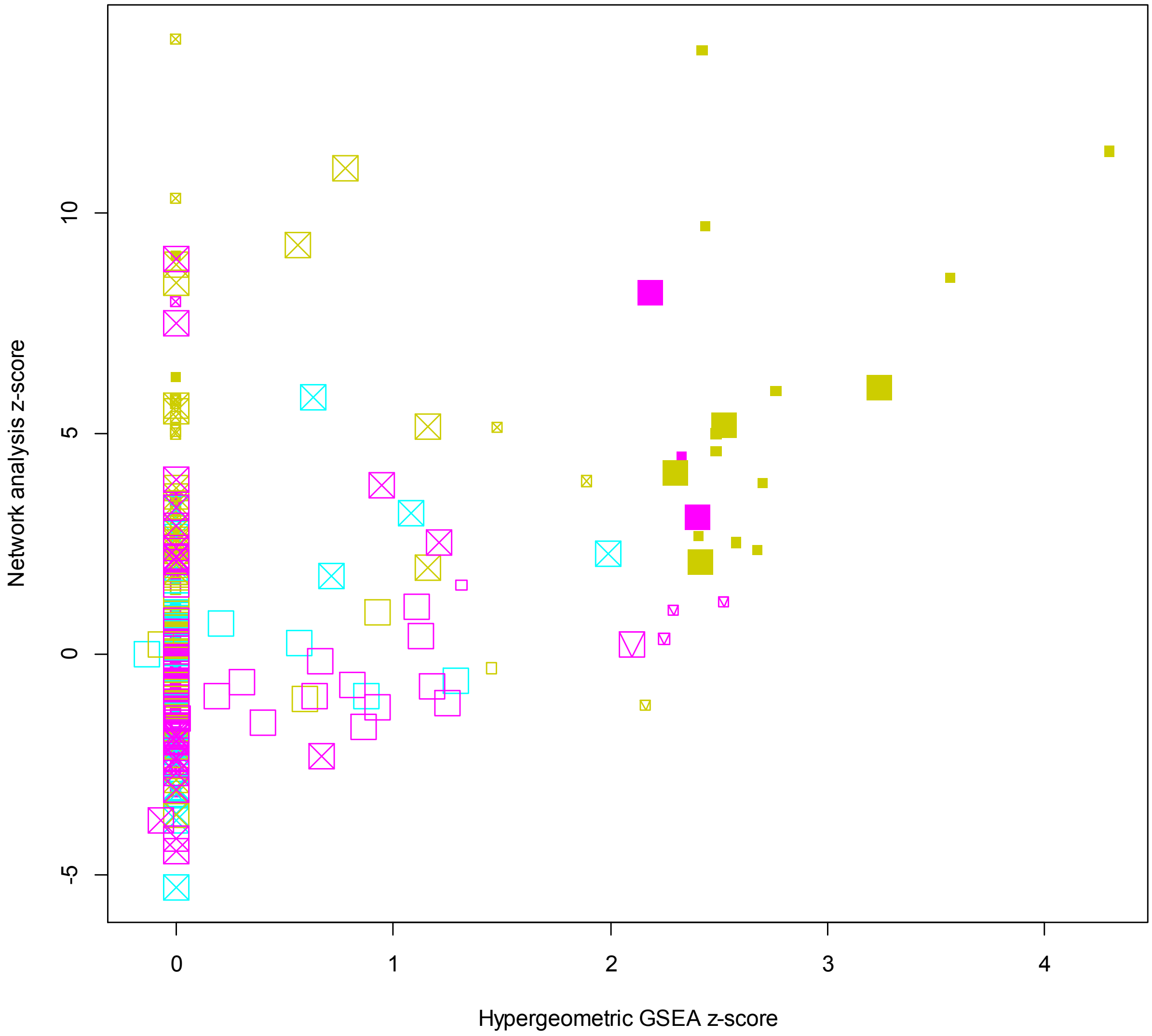

Figure S9. Comparative sensitivity of gene set-based enrichment analysis (GSEA) and network enrichment analysis (NEA) in regard of genes sets (single nucleotide variations, SNV and allelic imbalance, AI) from the three cell lines.
The analysis is the same as for Fig. 5, with the difference that all signaling and cancer pathways are shown. Gene sets with zero overlap (no shared genes) received GSEA z=0. Pairs of gene sets with zero overlap are seen as the vertical line at GSEA z=0.
Boxes:
small, AI gene sets;
large, SNV gene sets;
dark yellow, U251MG;
cyan, A431;
purple, U2OS.
empty, insignificant (FDR>0.1) by both GSEA and NEA;
checked with "V", significant only by GSEA;
checked with "X", significant only by GSEA;
filled, significant by both GSEA and NEA.

SUPPLEMENTARY TABLES

	U2OS	U251MG	A431
**DELETIONS**	1497	1405	1340
Exonic	54	28	25
Intronic	549	499	449
Intergenic	948	906	866
UTRs	68	55	48
**INVERSIONS**	149	85	51
Exonic	9	10	11
Intronic	39	22	11
Intergenic	101	53	29
**INTRA-CHROMOSOMAL TRANSLOCATIONS**			
Intergenic-intergenic	66	31	19
Intergenic-genic	7	0	1
Genic-genic	20	8	1
Exonic	5	3	0
**INTER-CHROMOSOMAL-TRANSLOCATIONS**			
Intergenic-intergenic	38	13	8
Intergenic-genic	9	5	3
Genic-genic	3	5	0
Exonic	2	1	0

Table S1. Breakdown of structural variations in U2OS, U251MG and A431 cell lines

Cell Line	Genes in gained regions	Number of Genes in lost regions	Number of genes completely lost
A431	4,877	91	12 (including CDKN2B)
U2OS	4,263	3,529	2
U251MG	4,954	24	18 (including CDKN2A, CDKN2B, CDKN2C)

Table S2. Of the 33,317 annotated gene features (RefSeq v51, (*60*), 4,877 and 91 were contained within amplified regions and lost regions respectively in A431 cells. 12 genes were completely lost, including CDKN2B. In U2OS, there were 4,263 and 3,529 genes that were contained within amplified and lost regions respectively. Only 2 genes (LCE3B and LCE3C) were completely

lost. U251-MG lost at least one copy of 24 genes and gained more copies of 4,954 genes. There were 18 genes completely lost, including CDKN2A, CDKN2B and CDKN2C.

SNVs	A431	U2OS	U251
Before filtering	2292	2340	2164
Fraction of supporting reads ≥ 0.45	1156	1061	1141
HapMap frequency (CEU) ≤ 0.05	453	412	462
RPKM (top 75th percentile)	156	158	183
S3APP calls	15	6	20
Causing aberrant transcript	0	1	1

Table S3. Filtering statistics of Splice Site SNVs

Allelically imbalanced gene	Gene name	Number of supporting SNVs	Alternative allele expressed?	Cell line
**BCLAF1**	Bcl-2-associated transcription factor 1	6	No	A431
**ZNF354C**	Zinc finger protein 354C	3	No	A431
**ZNF215**	Zinc finger protein 215	2	No	A431
**ZNF280C**	Zinc finger protein 280C	2	No	A431
**SLC16A14**	Monocarboxylate transporter 14	2	No	A431
**H2AFY2**	Core histone macro-H2A.2	1	Yes	A431
**PARM1**	Prostate androgen-regulated mucin-like protein 1	1	Yes	A431
**MTHFSD**	Methenyltetrahydrofolate synthase domain-containing protein	1	Yes	A431
**HDAC8**	Histone deacetylase 8	1	Yes	A431
**SMARCA1**	Probable global transcription activator SNF2L1	1	Yes	A431
**SPAG16**	Sperm-associated antigen 16 protein	1	Yes	A431
**TSC22D3**	TSC22 domain family protein 3	1	Yes	A431
**LY75**	Lymphocyte antigen 75	1	Yes	A431
**MAP2K3**	Dual specificity mitogen-activated protein kinase kinase 3	10	No	U251
**PTPRM**	Receptor-type tyrosine-protein phosphatase mu	3	No	U251
**MYO1B**	Myosin-1b	2	Yes	U251
**PTGFR**	Prostaglandin F2-alpha receptor	2	No	U251
**SH2D4A**	SH2 domain-containing protein 4A	2	Yes	U251
**KCNH1**	Potassium voltage-gated channel subfamily H member 1	1	Yes	U251
**GOLGA8A**	golgi autoantigen, golgin subfamily a, 8A; golgi autoantigen, golgin subfamily a, 8A	4	Yes	U2OS

**RBMX**	Heterogeneous nuclear ribonucleoprotein G	3	No	U2OS
**Map2k3**	mitogen-activated protein kinase kinase 3	3	No	U2OS
**PRKRA**	Interferon-inducible double stranded RNA-dependent protein kinase activator A	2	No	U2OS
**CCDC144NL**	Putative coiled-coil domain-containing protein 144 N-terminal-like	2	No	U2OS
**MKX**	mohawk homeobox	2	Yes	U2OS
**TUBA3E**	tubulin, alpha 3e	1	Yes	U2OS
**CMBL**	Carboxymethylenebutenolidase homolog	1	Yes	U2OS
**PTGIS**	prostaglandin I2 (prostacyclin) synthase	1	Yes	U2OS
**IFT172**	Intraflagellar transport protein 172 homolog	1	Yes	U2OS

Table S4. List of allelically imbalanced genes in all cell lines.

	**A431**	**U2OS**	**U251**
Before filtering	8479	8867	7951
Fraction of supporting reads ≥ 0.45	3994	3632	3803
HapMap freq (CEU) ≤ 0.05	1045	879	956
Polyphen2 FDR ≤ 0.1	256	188	240
Polyphen2 FDR ≤ 0.05	113	87	107
Base RNA coverage ≥ 5	57	51	54

Table S5. Filtering statistics of non-synonymous coding SNVs.

**Cell line**	**Network statistic**	**Gene symbol**	**No. of links observed in the actual network between the gene and other SNV genes in the same cell line**	**No. of links expected by chance**	**Standard deviation**	**Z-score**
**A431**	ind	FBXW7	1832	937.00	42.919	**20.8535**
**A431**	ind	KIAA0319	1560	723.00	43.331	**19.3165**
**A431**	dir	NRP1	1	0.10	0.316	**2.8460**

**A431**	dir	RNF40	2	0.30	0.483	**3.5193**
**A431**	ind	RNF43	248	124.20	15.245	**8.1209**
**A431**	dir	SUV420H1	1	0.10	0.316	**2.8460**
**A431**	dir	TNKS1BP1	1	0.10	0.316	**2.8460**
**A431**	ind	WDR47	1576	642.20	59.303	**15.7463**
**U2OS**	ind	CAD	1274	749.20	52.849	**9.9301**
**U2OS**	dir	CAD	4	1.00	1.054	**2.8460**
**U2OS**	dir	MTHFD1	2	0.40	0.516	**3.0984**
**U2OS**	ind	MTHFD1	720	368.60	22.609	**15.5427**
**U2OS**	ind	NUP93	1098	567.40	29.934	**17.7257**
**U2OS**	ind	PKMYT1	790	406.40	19.973	**19.2056**
**U2OS**	ind	PRIM2	648	327.20	34.282	**9.3575**
**U2OS**	ind	TOP1MT	126	49.60	8.682	**8.7998**
**U2OS**	ind	WDHD1	606	256.00	22.568	**15.5084**
**U251**	ind	CKAP2L	1222	342.60	27.520	**31.9544**
**U251**	dir	EIF2AK1	3	0.30	0.483	**5.5895**
**U251**	ind	EIF2AK1	372	177.40	26.982	**7.2121**
**U251**	ind	EIF2AK4	752	441.80	29.431	**10.5399**
**U251**	ind	FANCI	1554	471.00	23.462	**46.1606**
**U251**	dir	FANCI	3	0.60	0.516	**4.6476**
**U251**	dir	LAMA4	1	0.10	0.316	**2.8460**
**U251**	dir	LAMB1	1	0.10	0.316	**2.8460**
**U251**	ind	MCM3	2242	869.80	45.328	**30.2727**
**U251**	ind	NAT10	1108	571.00	26.887	**19.9728**
**U251**	dir	NCOA4	1	0.10	0.316	**2.8460**
**U251**	ind	PRIM2	1080	330.00	20.133	**37.2525**
**U251**	dir	PRIM2	3	0.60	0.699	**3.4325**
**U251**	ind	PVRL3	368	241.20	19.211	**6.6003**
**U251**	ind	QSER1	108	34.00	10.832	**6.8316**
**U251**	ind	SPEG	162	61.40	9.383	**10.7213**
**U251**	dir	SPEG	2	0.10	0.316	**6.0083**

Table S6. Most likely driver point mutations, i.e.SNV genes that are functionally connected to genes carrying potentially damaging SNVs in the same cell line, based on interaction network analysis. “ind” refers to indirect and “dir” refers to direct statistics counting connections between the genes and other SNV genes taken as a group.

Cell line	Number of genes with SNVs	Copy number altered gene	Number of network links between CNA gene and SNV genes	Number of links expected by chance	Standard deviation	Z-score
**U2OS**	51	MCM7	7	2	1.414	3.5355
**U2OS**	51	TYMS	6	0.9	0.738	6.9118
**U2OS**	51	MCM3	6	1.9	1.595	2.5703
**U2OS**	51	AURKB	5	0.5	0.527	8.5381
**U2OS**	51	AIFM1	5	0.7	0.675	6.3709
**U2OS**	51	AHCY	5	1.1	0.738	5.2855
**U2OS**	51	RRM2	5	1.5	0.707	4.9497
**U2OS**	51	KPNA2	5	1	1.054	3.7947
**U2OS**	51	MCM5	5	1.6	1.174	2.8966
**U2OS**	51	POLD2	4	0.4	0.516	6.9714
**U2OS**	51	EIF3B	4	0.8	0.789	4.0567
**U2OS**	51	KIF23	4	1.2	0.789	3.5496
**U2OS**	51	TRIP13	4	1.2	0.919	3.047
**U2OS**	51	CCNB2	4	1.3	0.949	2.846
**U2OS**	51	CAD	4	1.2	1.033	2.7111
**U2OS**	51	UBE2C	4	1.7	0.949	2.4244
**U2OS**	51	ACTG1	4	1.7	1.16	1.9836
**U2OS**	51	ARFGAP1	3	0.1	0.316	9.1706
**U2OS**	51	CCNE2	3	0.1	0.316	9.1706
**U2OS**	51	NEDD1	3	0.1	0.316	9.1706
**U2OS**	51	LAS1L	3	0.2	0.422	6.6408
**U2OS**	51	NUP155	3	0.3	0.483	5.5895
**U2OS**	51	ABCF2	3	0.4	0.516	5.0349
**U2OS**	51	KIF4A	3	0.6	0.516	4.6476
**U2OS**	51	ERCC6L	3	0.2	0.632	4.4272
**U2OS**	51	MOGS	3	0.3	0.675	4.0003
**U2OS**	51	CHAF1B	3	0.4	0.699	3.7185
**U2OS**	51	ATAD2	3	0.5	0.707	3.5355
**U2OS**	51	EIF5A	3	0.5	0.707	3.5355
**U2OS**	51	TIA1	3	0.8	0.632	3.4785
**U2OS**	51	NCAPH	3	0.7	0.675	3.4077
**U2OS**	51	PLK1	3	0.7	0.675	3.4077
**U2OS**	51	PBK	3	1.1	0.568	3.3472
**U2OS**	51	EBNA1BP2	3	1	0.667	3

**U2OS**	51	EIF2S3	3	0.6	0.843	2.846
**U2OS**	51	IMPDH1	3	0.9	0.738	2.846
**U2OS**	51	VRK1	3	0.9	0.738	2.846
**U2OS**	51	EVPL	3	0.7	0.823	2.7937
**U2OS**	51	PSMC3	3	0.7	0.823	2.7937
**U2OS**	51	TUBA3D	3	0.7	0.823	2.7937
**U2OS**	51	PNPT1	3	0.8	0.789	2.789
**U2OS**	51	ACTR3	3	0.7	0.949	2.4244
**U2OS**	51	ZWINT	3	0.7	0.949	2.4244
**U2OS**	51	ELAC2	3	0.9	0.876	2.3984
**U2OS**	51	PSMD13	3	0.9	0.876	2.3984
**U2OS**	51	NHP2L1	3	0.8	0.919	2.3941
**U2OS**	51	DDX18	3	1.1	0.876	2.17
**U2OS**	51	EXOSC4	3	1.1	0.876	2.17
**U2OS**	51	PES1	3	1.1	0.876	2.17
**U2OS**	51	NCAPD2	3	0.9	0.994	2.1118
**U251**	54	SMC4	8	2.6	2.413	2.2379
**U251**	54	RFC4	7	1.6	1.174	4.6005
**U251**	54	CLSPN	5	1	0.471	8.4853
**U251**	54	HELLS	5	0.3	0.675	6.9635
**U251**	54	FANCD2	5	0.4	0.699	6.5789
**U251**	54	DHX15	5	0.9	0.738	5.5566
**U251**	54	KIF23	5	0.7	0.823	5.2231
**U251**	54	KIF11	5	0.5	0.972	4.6305
**U251**	54	CKS1B	5	1	1.054	3.7947
**U251**	54	ECT2	5	1	1.054	3.7947
**U251**	54	CCNB2	5	1.5	1.269	2.7574
**U251**	54	KIF14	4	0.7	0.483	6.8316
**U251**	54	CCDC99	4	0.5	0.707	4.9497
**U251**	54	NUSAP1	4	0.5	0.707	4.9497
**U251**	54	POLG	4	0.5	0.707	4.9497
**U251**	54	ATAD2	4	0.6	0.699	4.8627
**U251**	54	CDCA8	4	0.6	0.699	4.8627
**U251**	54	NDC80	4	1	0.667	4.5
**U251**	54	TYMS	4	1	0.667	4.5
**U251**	54	KIF15	4	0.9	0.738	4.2013
**U251**	54	ZWINT	4	0.9	0.738	4.2013
**U251**	54	KIF2C	4	0.6	0.843	4.0319
**U251**	54	NUF2	4	0.6	0.843	4.0319
**U251**	54	KIF20A	4	0.7	0.823	4.0084
**U251**	54	SFPQ	4	0.7	0.823	4.0084

**U251**	54	SERBP1	4	1	0.816	3.6742
**U251**	54	CDC25C	4	0.9	0.876	3.5404
**U251**	54	DTL	4	0.9	0.876	3.5404
**U251**	54	PRC1	4	0.9	0.876	3.5404
**U251**	54	PSMA4	4	0.8	0.919	3.4823
**U251**	54	SHC1	4	1.1	0.876	3.312
**U251**	54	CENPH	4	0.8	1.033	3.0984
**U251**	54	CCNB1	4	1.4	0.843	3.0832
**U251**	54	TCERG1	4	1.3	1.059	2.5487
**U251**	54	CSK	4	1.2	1.229	2.2778
**U251**	54	TRA2B	4	1.5	1.179	2.1213
**U251**	54	NUP160	3	0.2	0.422	6.6408
**U251**	54	PDS5A	3	0.4	0.516	5.0349
**U251**	54	WHSC1	3	0.4	0.516	5.0349
**U251**	54	SF3B4	3	0.6	0.516	4.6476
**U251**	54	TACC3	3	0.6	0.516	4.6476
**U251**	54	UBE2T	3	0.6	0.516	4.6476
**U251**	54	HMMR	3	0.4	0.699	3.7185
**U251**	54	GAK	3	0.5	0.707	3.5355
**U251**	54	UBR1	3	0.8	0.632	3.4785
**U251**	54	USP1	3	0.8	0.632	3.4785
**U251**	54	YES1	3	0.8	0.632	3.4785
**U251**	54	AP2M1	3	0.6	0.699	3.4325
**U251**	54	CENPL	3	0.6	0.699	3.4325
**U251**	54	LMNB1	3	0.6	0.699	3.4325
**U251**	54	CD44	3	0.7	0.675	3.4077
**U251**	54	PRIM2	3	0.7	0.675	3.4077
**U251**	54	CDC20	3	1.2	0.632	2.846
**U251**	54	FANCI	3	0.7	0.823	2.7937
**U251**	54	TOP2B	3	0.9	0.876	2.3984
**U251**	54	ACTL6A	3	1.1	0.876	2.17
**U251**	54	HSPA4	3	1.1	0.876	2.17
**U251**	54	EIF3M	3	1	0.943	2.1213
**U251**	54	MKI67	3	1	0.943	2.1213
**A431**	57	TIA1	7	1.8	1.317	3.9497
**A431**	57	RFC4	6	2.4	1.174	3.067
**A431**	57	SMC4	6	2.2	1.476	2.575
**A431**	57	CEBPZ	4	0.9	0.568	5.4611
**A431**	57	PYGL	4	0.9	0.738	4.2013
**A431**	57	ACIN1	4	1	0.943	3.182
**A431**	57	ATP6V1H	4	0.8	1.135	2.8187

**A431**	57	NUP98	4	1.5	1.269	1.9696
**A431**	57	PRPF19	4	1.5	1.269	1.9696
**A431**	57	HNRPLL	3	0.1	0.316	9.1706
**A431**	57	IPO4	3	0.3	0.483	5.5895
**A431**	57	GCC2	3	0.4	0.699	3.7185
**A431**	57	HSP90AB1	3	1.3	0.483	3.5193
**A431**	57	EIF4A2	3	0.7	0.675	3.4077
**A431**	57	CAP2	3	0.6	0.843	2.846
**A431**	57	GDA	3	0.6	0.843	2.846
**A431**	57	RANBP2	3	0.8	0.789	2.789
**A431**	57	MSH2	3	1	0.816	2.4495
**A431**	57	FNDC3B	3	0.7	0.949	2.4244
**A431**	57	RTN3	3	1.4	0.699	2.2883
**A431**	57	MOGS	3	0.6	1.075	2.2326
**A431**	57	MKI67IP	3	1.1	0.876	2.17
**A431**	57	DLD	3	0.9	0.994	2.1118

Table S7. Most likely driver copy-number alterations, i.e.CNA genes that are functionally connected to genes carrying potentially damaging SNVs in the same cell line.
